# Value of gamma interferon enzyme-linked immunospot assay in the diagnosis of peritoneal dialysis-associated tuberculous peritonitis

**DOI:** 10.1007/s11255-021-02960-1

**Published:** 2021-07-14

**Authors:** Qiuxia Fan, Xiaoyan Huang, Jieyun Zhang, Yinan Sun, Zuying Xiong, Zibo Xiong

**Affiliations:** 1 Renal Division, Peking University Shenzhen Hospital, Futian district, Lianhua road 1120, Shenzhen, CN 518036 Guangdong China; 2 Guangdong Key Laboratory for Diagnosis and Treatment of Emerging Infectious Diseases, The Affiliated Shenzhen Third Hospital, Shenzhen, 518020 China

**Keywords:** Gamma interferon, Enzyme-linked immunospot experiments, Peritoneal dialysis, Tuberculous peritonitis

## Abstract

**Background:**

Tuberculous peritonitis is the most common form of extrapulmonary tuberculosis infection in peritoneal dialysis patients. However, diagnosing tuberculous peritonitis quickly and early has always been a challenge for nephrologists. *Mycobacterium tuberculosis* antigen-specific gamma interferon enzyme-linked immunospot (IFN-γ ELISPOT) assay has been widely used in the clinical diagnosis of tuberculous pleurisy and peritonitis, but its use has not been reported for uremia.

**Methods:**

This study mainly verified the feasibility of using the *M. tuberculosis* antigen-specific IFN-γ ELISPOT assay in the diagnosis of continuous ambulatory peritoneal dialysis (CAPD) patients with tuberculous peritonitis. Taking *M. tuberculosis* culture as the gold standard, the IFN-γ ELISPOT assay was used to analyze peripheral blood and peritoneal dialysis fluid of patients, and the receiver operating characteristic (ROC) curves in patients with tuberculous peritonitis (TBP) or non-tuberculous peritonitis (NTBP) were analyzed.

**Results:**

The area under the receiver operating characteristic curve (AUC) was 0.927 (95% CI 0.816–1.000, *P* = 0.001) for the ELISPOT assay with peritoneal fluid mononuclear cells (PFMC), which was higher than that for the ELISPOT assay with peripheral blood mononuclear cells (PBMC) (0.825, 95% CI 0.6490–1.000, *P* = 0.011). The cutoff value for the diagnosis of TBP was 40 spot-forming cells (SFCs)/2 × 10^5^ for the ELISPOT with PBMC, with a sensitivity of 55.6%, a specificity of 92.3%, and a diagnostic efficiency of 77.3%. The cutoff value for the diagnosis of TBP was 100 SFCs/2 × 10^5^ for the ELISPOT on PFMC, with a sensitivity, specificity, and diagnostic efficiency 77.8%, 84.6%, and 81.8%, respectively. Parallel and serial testing algorithms appeared more accurate than single ELISPOT assays with PBMC, but ELISPOT assays with PFMC.

**Conclusions:**

The IFN-γ release test can be used for the early diagnosis of CAPD-related TBP; compared with peripheral blood, peritoneal fluid may be a more effective and accurate medium to diagnose CAPD complicated with tuberculous peritonitis.

## Introduction

Patients with end-stage renal disease (ESRD) undergoing chronic dialysis are 6–25 times more likely to develop tuberculosis (TB) than the general population, mainly because of impaired cellular immunity, anemia, malnutrition, etc. [1–4]. Tuberculous peritonitis (TBP) is the most common form of extrapulmonary tuberculosis infection in peritoneal dialysis patients [5]. However, diagnosing active TB infection can be challenging. *M. tuberculosis* (liquid and solid) culture remains the gold standard diagnostic test, but it can take up to 10 weeks to become positive, introducing delays in diagnosis and treatment as well as associated increased morbidity [6]. Therefore, early and rapid diagnosis of tuberculous peritonitis has always been a challenge for nephrologists. T cell interferon gamma (IFN-γ) release assays (IGRAs) are now used to diagnose latent TB infection (LTBI) [7–9]and include a whole-blood gamma interferon (IFN-γ) enzyme-linked immunosorbent assay (FERQuanti ON-TB Gold in-tube (QFT-G); Cellestis Ltd, Victoria, Australia) and an enzyme-linked immunospot (ELISPOT) assay (T-SPOT.TB; Oxford Immunotec, Oxfordshire, United Kingdom). *M. tuberculosis* antigen-specific gamma interferon (IFN-γ) production by peripheral blood mononuclear cells (PBMC) was determined by using an in-house enzyme-linked immunospot (ELISPOT) assay by Institute of Hepatology of Shenzhen Third Hospital [10]. The researchers developed and evaluated an in-house IFN-γ ELISPOT assay for the diagnosis of active TB, compared the performance of their ELISPOT assay with that of the commercial QFT-IT assay and analyzed the influence of clinical manifestations on the accuracy of the ELISPOT assay. In this study, we evaluated the diagnostic performance of an *M. tuberculosis* antigen-specific IFN-γ ELISPOT assay in continuous ambulatory peritoneal dialysis (CAPD) patients with tuberculous peritonitis.

## Materials and methods

### Study population

**Fig. 1:**
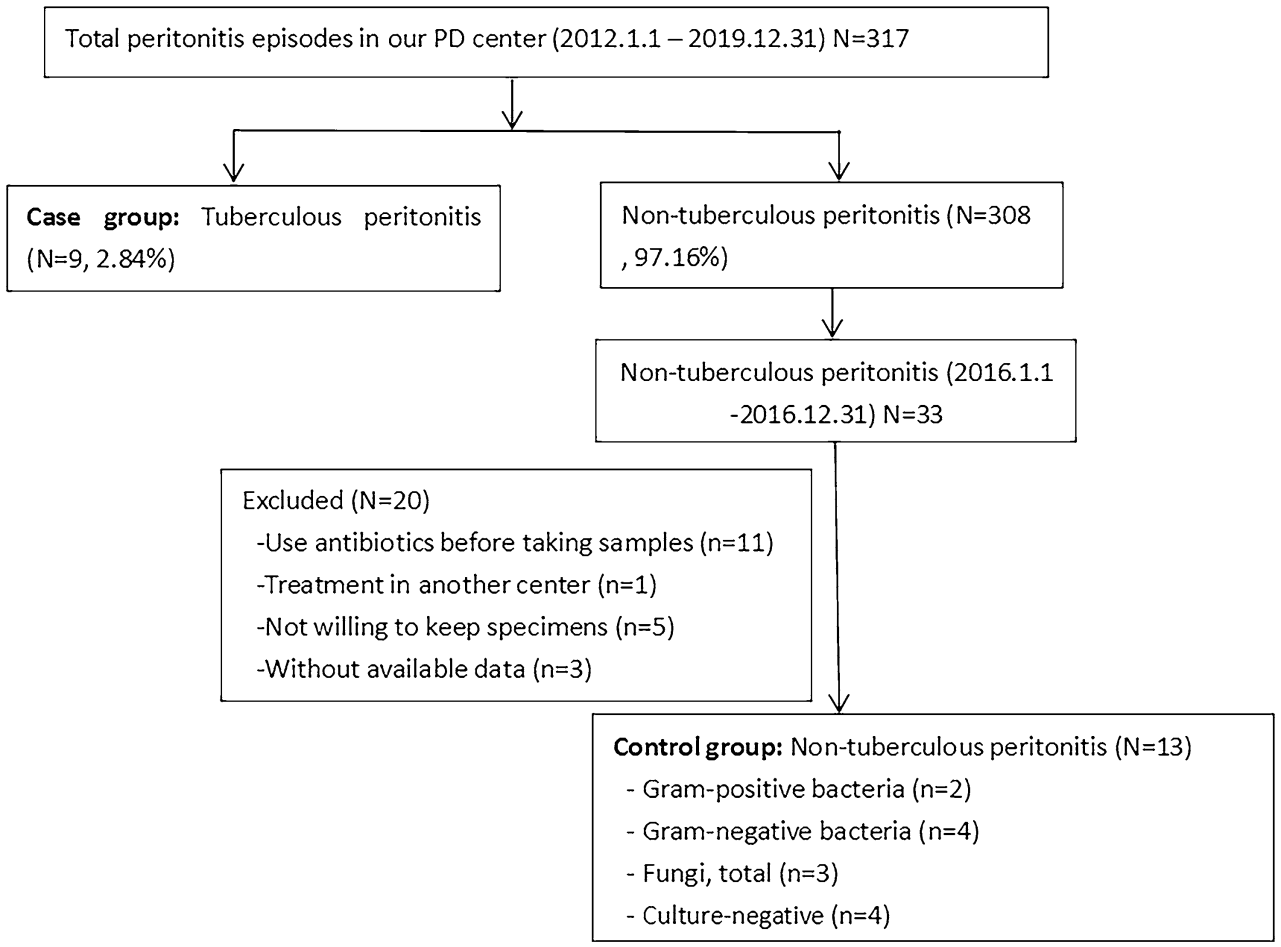
Flow chart of the study population. *TBP* tuberculous peritonitis, *NTBP*, non-tuberculous peritonitis, *PBMC* peripheral blood mononuclear cells, *PFMC* peritoneal fluid mononuclear cells

A cross-sectional study was performed, in which 22 patients with peritonitis were enrolled at Peking University Shenzhen Hospital from January 2012 to December 2019. This study was approved by the Ethics Committee of Peking University Shenzhen Hospital. Written informed consent was obtained from all patients enrolled in this study. The incidence of peritonitis ranged from 0.102 to 0.225 episodes per patient years in our peritoneal dialysis (PD) center. The total number of peritonitis episodes in the center was 317, and *M. tuberculosis* accounted for 2.84% of all peritonitis cases. A total of 308 episodes (97.16%) had non-tuberculous peritonitis among all peritonitis cases. There were 33 patients with non-tuberculous peritonitis from January to December 2016. Of these patients, 20 patients were excluded from our study for the following reasons: antibiotics were used before taking samples (*n* = 11), treatment was provided in another center (*n* = 1), the patients were unwilling to keep specimens (*n* = 5), and data was unavailable (*n* = 3). Finally, 13 patients with bacterial or fungal peritonitis were enrolled as controls. Of the 13 patients with non-tuberculous peritonitis, 2 cases were caused by gram-positive bacteria, 4 were caused by gram-negative bacteria, and 3 were caused by fungi, and 4 patients had negative pathogenic bacterial cultures and were cured after routine antibiotic treatment (ceftazidime combined with cefazolin). The flow chart of the study population is shown in Fig. 1. Patients with HIV infection and active tuberculosis in other locations were excluded.

### Diagnostic criteria

Peritoneal dialysis-related peritonitis was always diagnosed when at least 2 of the following were present: (1) clinical features consistent with peritonitis, i.e., abdominal pain and/or cloudy dialysis effluent; (2) dialysis effluent white cell count > 100/μL or > 0.1 × 10^9^/L (after a dwell time of at least 2 h), with > 50% polymorphonuclear cells; and (3) positive dialysis effluent culture. The diagnosis of tuberculous peritonitis was confirmed if a patient’s dialysis effluent was positive for *M. tuberculosis* culture. According to symptoms, physical signs, the clinical course, and laboratory and imaging examination findings, patients with highly suspected tuberculous peritonitis could also be diagnosed if their PD effluent was culture-negative and improved after anti-tuberculosis treatment [11].

### PBMC and PFMC ELISPOT assays

Peripheral blood and peritoneal fluid were obtained before the treatment of peritonitis patients, at this time, patients often have clinical manifestations such as abdominal pain, fever, cloudy dialysis effluent, and excessive number of peritoneal fluid cells. Ten milliliters of peripheral blood were collected from the participants, and peripheral blood mononuclear cells (PBMC) were obtained from whole blood by centrifugation over a Ficoll Hypaque density gradient (Ficoll-Paque Plus; Amersham Biosciences). Cells were resuspended in Lympho-Spot medium (U-CyTech Bioscience, the Netherlands). At the same time, 50–100 mL of peritoneal fluid that remained within the intraperitoneal cavity for no less than 2 h was collected to obtain PFMC by centrifugation at 300×*g* for 5 min. It was necessary to reach 2 × 10^5^ PFMC/well for a total of four wells, and the number of monocytes required was 8 × 10^5^, which could be obtained by centrifugation of 50–100 mL of peritoneal dialysis fluid. The sediment was suspended in 5 mL of red blood cell lysis solution (MiltenyiBiotec GmbH, Germany), allowed to stand for 1 min at room temperature, and then mixed with 5 mL of RPMI 1640 containing 10% fetal bovine serum (FBS). This suspension was centrifuged at 300×*g* for 5 min, and the cells were resuspended in 5 mL of RPMI 1640 containing 10% FBS. Ten microliters of this suspension were mixed with 90 μL of trypan blue solution to evaluate the viability and to count cells. The cells were resuspended in Lympho-Spot medium (U-CyTech Biosciences, the Netherlands) at a final concentration of 2 × 10^6^ cells/mL. PBMC or PFMC (2 × 10^5^/well) were seeded in duplicate in 96-well plates (MultiScreen-IP; Millipore) precoated with an anti-IFN-γ capture monoclonal antibody (eBioscience). Cells were stimulated with the *Mycobacterium tuberculosis*-specific antigen early secretory antigenic target 6 (ESAT-6) protein (provided by Haiying Liu, Chinese Academy of Medical Sciences) at 10 μg/mL for 24 h at 37 ℃ and with 5% CO_2_. PBMC and PFMC in medium alone or stimulated with phytohemagglutinin (Sigma) at 2.5 μg/mL were used as negative or positive controls, respectively. Biotinylated anti-IFN-γ detection monoclonal antibodies (eBioscience) were added for 1 h, followed by the addition of streptavidin–alkaline phosphatase conjugate (Pierce Biotechnology), which was incubated for 1 h. After a wash step, tetrazolium-BCIP (5-bromo-4-chloro-3-indolyl phosphate; Sigma, USA) chromogenic substrate was added. The individual spots were counted using an automated image analysis ELISPOT reader system (BioReader 4000 Pro-X; Biosys, Germany) [10, 12, 13].

### Statistical analysis

Statistical analyses were performed using IBM SPSS for Windows, version 23.0 (SPSS); the MedCalc software package, version 18.2.1.0 (MedCalc Software); and GraphPad Prism, version 8.0.1 (GraphPad, La Jolla, CA). Continuous variables were compared using the Mann–Whitney *U* test or Student’s *t* test, and Fisher’s exact test was used to compare categorical variables. *P* values < 0.05 were considered significant. Diagnostic performance was expressed in terms of sensitivity, specificity, positive predictive value, negative predictive value, positive likelihood ratio, and negative likelihood ratio.

## Results

### Patient characteristics

A total of 22 patients with peritonitis were qualified and recruited for this study. Nine patients were diagnosed with TBP. Among them, 8 were positive, and 1 was negative in dialysate by *M. tuberculosis* culture; the patient with a negative culture was diagnosed by clinical manifestations, and effective anti-tuberculosis treatment was performed after 2–4 weeks. The remaining 13 patients were confirmed to have peritonitis that were not caused by *M. tuberculosis* infection (NTBP). The clinical characteristics of the patients are summarized in Table 1. Overall, there were significant differences in the spot number of ESAT-6-induced IFN-γ production in peritoneal fluid and the spot number in peripheral blood, and peritoneal fluid was 4–9 times higher than that in peripheral blood. There was a higher percentage of TB patients with a previous infection history than of NTB patients, and there was no difference in serum creatinine, blood urea nitrogen, albumin (ALB) or hemoglobin (HGB) levels.

**Table 1:** Baseline clinical characteristics of peritoneal dialysis-associated peritonitis patients

Characteristic	TBP (*n* = 9)	NTBP (*n* = 13)	*P* value
Gender (% male)	7 (77.8%)	4 (30.8%)	0.080^b^
Age (years)	57 ± 11.44	56.54 ± 14.85	0.938^a^
PD duration (years)	3.06 ± 2.38	3.63 ± 2.68	0.611^a^
Diabetes mellitus (%)	3 (33.3%)	2 (15.4%)	0.323^b^
Previous history of TB (%)	4 (44.4%)	0 (0%)	0.017^b^
The main leukocytes from PF are mononuclear cells (%)	2 (22.2%)	0 (0%)	0.156^b^
ESAT-6-induced IFN-γ production from PB (SFC/2 × 10^5^ PBMC)	41 (17.5–91)	8 (2.5–36)	0.011^c^
ESAT-6-induced IFN-γ production from PF (SFC/2 × 10^5^ PBMC)	385 (201–393)	35 (2–54)	0.001^c^
WBC from PB (× 10^9^/L)	7.9 ± 4.68	9.17 ± 3.76	0.491^a^
WBC from PF (× 10^6^/L)	315 (113–735)	1270 (166–6483.5)	0.095^c^
Hemoglobin (g/L)	99.0 ± 23.7	93.62 ± 15.5	0.525^a^
Serum creatinine (μmol/L)	810 (757.65–1097.4)	715 (620–825)	0.089^c^
Blood urea nitrogen (mmol/L)	15.15 ± 1.60	12.83 ± 4.35	0.144^a^
Calcium and phosphorus product (mg^2^/dL^2^)	33.97 (29.12–47.63)	32.1 (26.64–44.4)	0.483^c^
Albumin (g/L)	27.50 ± 6.15	27.27 ± 5.33	0.954^a^
Parathyroid hormone (pmol/L)	18.2 (11.3–31)	19.7 (10.1–42.7)	0.92^c^

*TB* tuberculosis, *PBMC* peripheral blood mononuclear cells, *PFMC* peritoneal fluid mononuclear cells

^a^Independent sample *t* test

^b^Fisher exact test

^c^Mann-Whitney test

### Results of ELISPOT assays

**Fig. 2:**
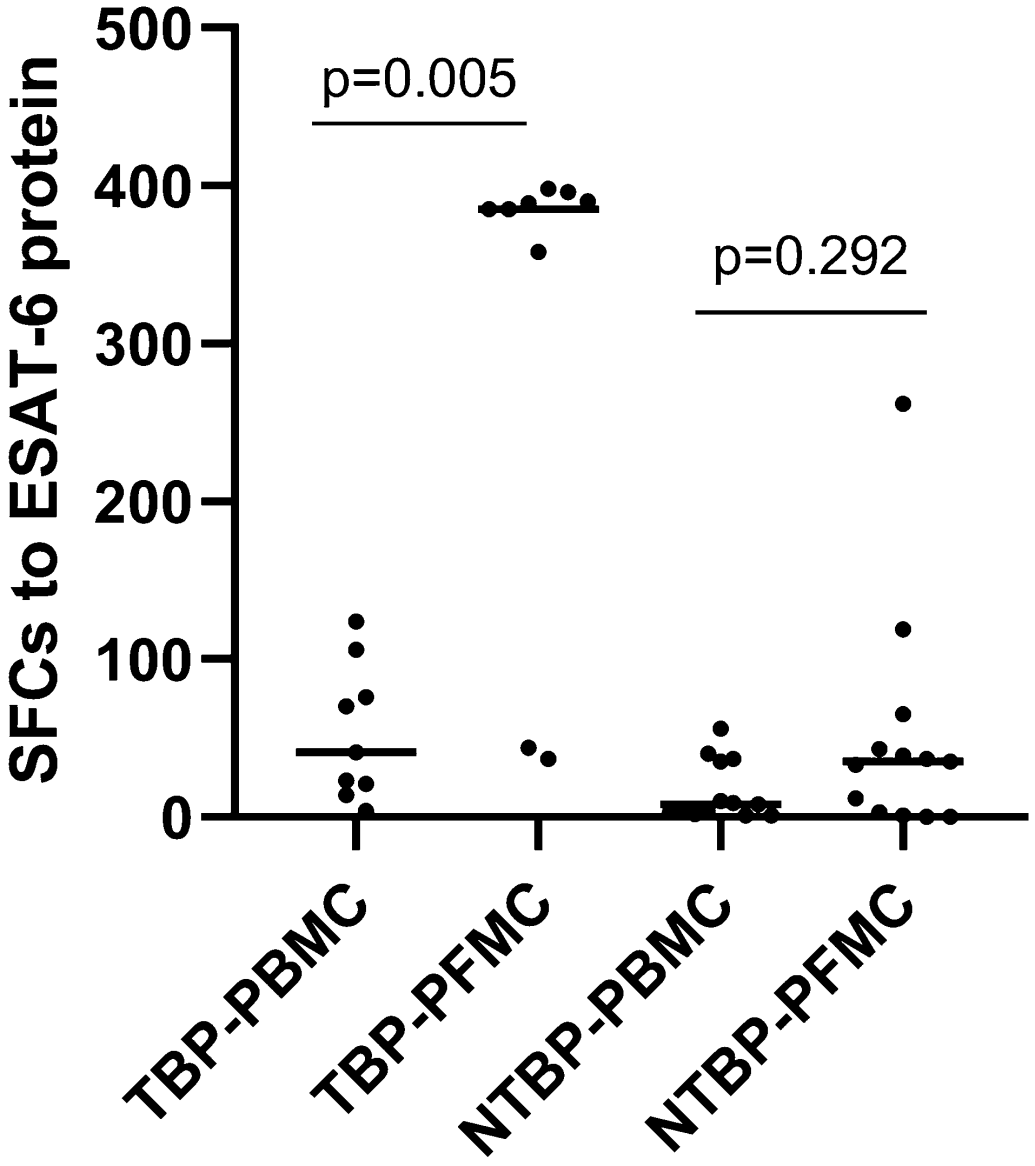
*M. tuberculosis* antigen-specific IFN-γ responses are enriched in PFMC over PBMC from patients with TBP. IFN-γ responses to ESAT-6 protein were determined in PBMC and PFMC from patients with TBP and from NTBP patients. Each spot represents an individual data point. *SFCs* spot-forming cells, *ESAT-6 protein* *mycobacterium tuberculosis*-specific antigen early secretory antigenic target 6

ELISPOT assays using PBMC and PFMC were performed on samples from all 22 subjects; 5 (55.6%) out of 9 patients with TBP were positive by the PBMC ELISPOT assay, and 7 (77.8%) out of 9 were positive by the PFMC ELISPOT assay. Among the controls, the ELISPOT assay was positive for one (7.7%) out of 13 NTBP patients when performed on PBMC and two (15.4%) out of 13 when performed on PFMC. The numbers of SFCs in response to ESAT-6 protein among PBMC are in good correlation with those among PFMC (*r*_s_ = 0.579, *P* = 0.005), indicating that the protein can efficiently stimulate IFN-γ production in an ELISPOT assay in both PBMC and in PFMC. We found that the levels of antigen-specific IFN-γ-producing cells were significantly increased among PFMC compared with those among matched PBMC in TBP patients, the median were 385 and 41 SFCs/2 × 10^5^, respectively (*P* = 0.005). As expected, the numbers of antigen-specific IFN-γ-producing cells were not different between PFMC and PBMC from NTBP patients, the median were 35 and 8 SFCs/2 × 10^5^, respectively (*P* = 0.292) (Fig. 2).

**Fig. 3:**
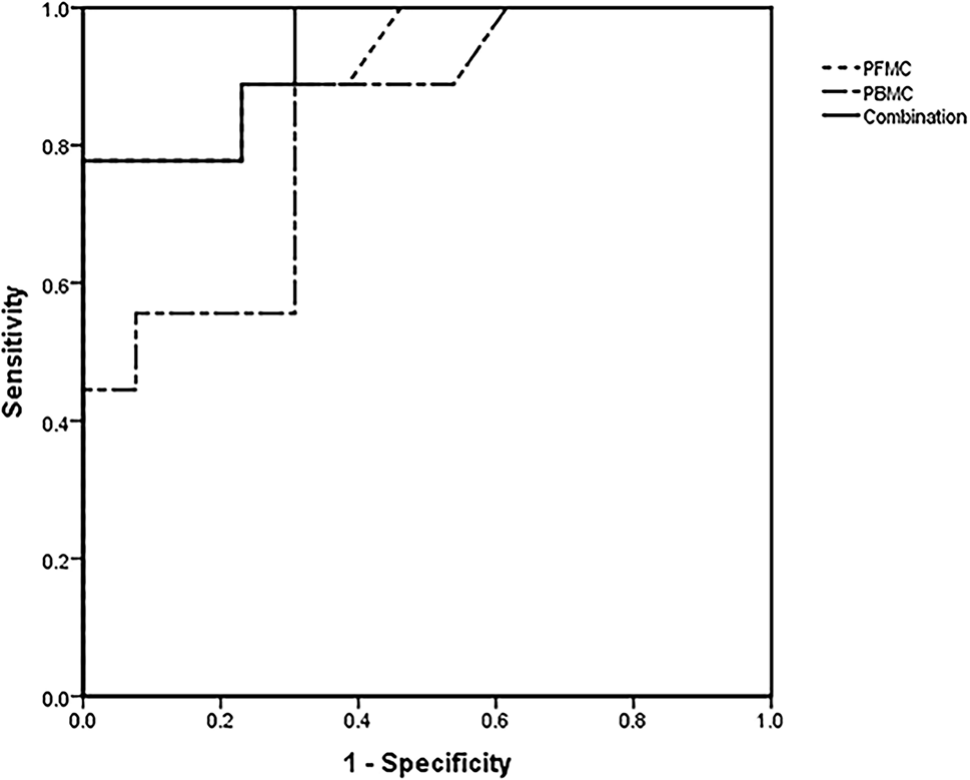
ROC curves for the ELISPOT assays with PFMC and PBMC from patients with tuberculous peritonitis or non-tuberculous peritonitis. The *M. tuberculosis* antigen-specific IFN-γ response in PFMC ELISPOT assays is useful for the diagnosis of TBP. The AUC was 0.927 (95% CI 0.816–1.000, *P* = 0.001) for the ELISPOT assay with PFMC, which was higher than that of the ELISPOT assay with PBMC (0.825, 95% CI 0.6490–1.000, *P* = 0.011). The AUC of the combination of PBMC and PFMC increased the c-statistic from 0.927 to 0.94. *PBMC* peripheral blood mononuclear cells, *PFMC* peritoneal fluid mononuclear cells, *ROC* receiver operating characteristic, *AUC* area under the receiver operating characteristic curve. The AUC of the combination of the ELISPOT assays with PFMC and PBMC to diagnose TBP was calculated with ROC curve analysis. The cutoff values of PFMC and PBMC in identifying TBP were 100 and 40 SFCs/2 × 10^5^, respectively, which were obtained according to Chen’s study

Receiver operating characteristic (ROC) analysis demonstrated that PFMC ELISPOT assays had higher areas under the curve (AUCs) than PBMC ELISPOT assays (Fig. 3). The AUC was 0.927 (95% CI 0.816–1.000, *P* = 0.001) for the ELISPOT assay with PFMC, which was higher than that for the ELISPOT on PBMC (0.825, 95% CI 0.6490–1.000, *P* = 0.011). The cutoff value for the diagnosis of TBP was 40 spot-forming cells (SFCs)/2 × 10^5^ for the ELISPOT assay with PBMC, with a sensitivity of 55.6%, a specificity of 92.3%, and a diagnostic efficiency of 77.3%. The positive predictive value was 83.3%, with a negative predictive value of 75%, a positive likelihood ratio of 7.2, and a negative likelihood ratio of 0.48. The cutoff value for the diagnosis of TBP was 100 SFCs/2 × 10^5^ for the ELISPOT assay with PFMC, with sensitivity, specificity, diagnostic efficiency, PPV, NPV, LR + , and LR − of 77.8%, 84.6%, 81.8%, 77.8%, 84.6%, 5.05 and 0.26, respectively. Thus, the performance of the PFMC ELISPOT assay for the diagnosis of TBP is significantly better than that of the PBMC ELISPOT assay. Parallel and serial testing algorithms appeared more accurate than the single ELISPOT assay with PBMC, but ELISPOT assay with PFMC. The parallel testing increased the sensitivity of the ELISPOT assay with PFMC from 77.8 to 88.9%, but the specificity was decreased from 84.6 to 76.9%. Serial testing increased the specificity of the ELISPOT assay with PFMC from 87.1 to 100%, but the sensitivity was decreased from 55.6 to 44.4% (Table 2).

**Table 2:** Diagnostic accuracy estimates for the ESAT-6 interferon-γ ELISPOT assay with PFMC and PBMC

	Sensitivity, %	Specificity, %	Diagnostic efficiency, %	PPV, %	NPV, %	LR +	LR −
ELISPOT with PFMC	77.8	84.6	81.8	77.8	84.6	5.05	0.26
ELISPOT with PBMC	55.6	92.3	77.3	83.3	75.0	7.20	0.48
ELISPOT with PBMC & PFMC (parallel)	88.9	76.9	81.8	72.7	90.9	3.85	0.14
ELISPOT with PBMC & PFMC (serial)	44.4	100	77.3	100	72.0	N/A	0.56

*PPV* positive predictive value, *NPV* negative predictive value, *LR + * positive likelihood ratio, *LR −* negative likelihood ratio, *PBMC* peripheral blood mononuclear cells, *PFMC* peritoneal fluid mononuclear cells, *N/A* not applicable

## Discussion

Peritoneal dialysis-related tuberculous peritonitis is the most common form of extrapulmonary tuberculosis infection in CAPD patients [5]. Diagnosis can often be challenging, due to the indolent nature of the presentation of TB peritonitis and relative unfamiliarity with the infection, and even when clinically suspected, diagnosing active TB infection is not easy. Mycobacterial culture remains the gold standard laboratory diagnostic method for TB peritonitis, but even with modern liquid culture techniques, a positive result may require up to 10 weeks of incubation [6]. The positive period of tuberculosis culture dialysis fluid of 8 cases was 6–10 weeks. Second, the acid-fast bacillus smear of peritoneal dialysis fluid is not sensitive. Only 2 of 9 cases of TBP were positive for acid-fast staining, accounting for 22.2%, so the rate of missed diagnosis was high. Furthermore, due to the low delayed hypersensitivity in ESRD patients, the positive rate of the tuberculin purified protein derivative (PPD) test in dialysis patients was 15% on average. Due to extensive vaccination with the Bacillus Calmette-Guerin (BCG) vaccine, the PPD test has lost its diagnostic value and has no reference value in dialysis patients [14]. Additionally, the clinical symptoms of TBP in CAPD are fever, abdominal pain and turbid peritoneal dialysate, which are similar to those of non-tuberculous peritonitis and are not typical. This delay in diagnosis and treatment can result in increased morbidity and may have infection control implications. A delay in diagnosis in the presence of persistent symptoms may mean that the PD catheter has to be removed, adding to the treatment burden of the patient having to convert to hemodialysis. Therefore, it is difficult to diagnose and treat TBP at this stage, and the mortality rate is much higher than that of non-tuberculosis peritonitis [15]. The best way to improve the survival rate of patients is to find an effective method for the early diagnosis of TBP and provide timely treatment.

In 2009, Chen, Xinchun et al. developed an in-house IFN-γ enzyme-linked immunospot (ELISPOT) assay and evaluated its value in the diagnosis of active tuberculosis (TB) in Shenzhen, China [10]. Their study demonstrates that the IFN-γ ELISPOT assay is a useful adjunct to current tests for the diagnosis of active TB in China. They developed an independent antigen-specific IFN-γ ELISPOT kit for tuberculosis bacteria. Compared with results from a QFT-G kit, the positive rates were 75.0% and 78.1%, and the difference was insignificant (*P* > 0.05). The positive threshold of ESAT-6 in peripheral blood was 40 SFCs/2 × 10^5^. In our study, the optimal critical value of ESAT-6-induced spots in peripheral blood was 40 SFCs/2 × 10^5^, and the sensitivity and specificity were 85.7% and 84.6%, respectively, which were close to the values reported in Chen's study. This finding shows that the uremic population and the general population have the same diagnostic threshold for peripheral blood.

The early diagnosis of tuberculosis infection by IGRAs is mainly based on the detection of peripheral blood antigen-stimulated polymorphonuclear cells. However, the detection with peripheral blood cannot effectively distinguish active TB from latent TB. In CAPD patients with tuberculous peritonitis, the number of leukocytes in the exudate increased significantly. It is feasible to use exudate monocytes to assist in the diagnosis of tuberculous peritonitis. The design theory of this experiment explored the feasibility of exudate monocytes in an ELISPOT to assist in the diagnosis of CAPD patients with tuberculous peritonitis. Wilkinson KA et al. first proposed the feasibility of using ELISPOT assays with monocytes from pleural and ascitic fluid to diagnose tuberculous serositis. In patients with tuberculosis, the mean concentration (± SD) of antigen-specific, IFN-γ-producing SFCs in the ascitic fluid was approximately 17-fold ± sixfold greater than that in peripheral blood [16]. In our study, we also found that the number of IFN-γ-specific T cells in the peritoneal dialysis fluid of patients with tuberculous peritonitis was 4–9 times higher than that in their peripheral blood.

Previous studies by our collaborators found that when the detection threshold of a TB-specific IFN-gamma ELISPOT in pleural effusion was 100 SFCs/2 × 10^5^, the sensitivity and specificity for the diagnosis of tuberculous pleurisy were 95.7% and 100%, respectively [13]. Sung-Han Kim et al. demonstrated that an ELISPOT assay using PBMC and PFMC is a useful adjunct to the current tests for diagnosing abdominal TB [17]. One possible explanation for the results is that an inflamed serosal surface may inevitably allow circulating M. tuberculosis-specific lymphocyte migration in patients with inactive TB and latent TB infection. In our study, when the cutoff value of the recombinant antigen ESAT-6 in dialysis fluid was 100 SFCs/2 × 10^5^, the diagnostic sensitivity was 77.8%, and the specificity was 84.6%. The AUC for the ELISPOT assay with PFMC was higher than that of the ELISPOT assay with PBMC, and the AUC of the combination of PBMC and PFMC increased the c-statistic from 0.93 to 0.94. Therefore, compared with peripheral blood, peritoneal dialysate may be a more effective and accurate medium to diagnose CAPD complicated with tuberculous peritonitis, and the combination of peritoneal dialysate and peripheral blood may also be an effective choice. The ELISPOT technique for peritoneal dialysis fluid is expected to be a rapid method for the clinical diagnosis of tuberculous peritonitis, and it is worth popularizing and applying widely.

Whether catheter removal is necessary for tuberculous peritonitis is still controversial. The 2016 ISPD guidelines tend to support the notion of extubation when *M. tuberculosis* peritonitis occurs and to consider reintroduction after 6 weeks of anti-tuberculosis treatment. With an increasing number of reported cases without extubation, some scholars believe that peritoneal function is an important condition to consider to determine whether patients continue peritoneal dialysis, and studies have proven that tuberculosis bacteria cannot survive in peritoneal dialysis catheters. If patients can maintain a certain level of ultrafiltration and transport function, they may not be extubated. Tuberculosis infection is not a sufficient condition to terminate peritoneal dialysis [18]. In this study, 2 patients were not extubated and continued undergoing peritoneal dialysis, which also supports this point of view.

Our study has several strengths and shortcomings. The major strengths of this study include that we enrolled nine tuberculosis peritonitis patients in 8 years, which is not easy because CAPD-related TBP is a rare disease. Moreover, tuberculosis culture was used as the golden standard in diagnosing tuberculosis peritonitis, all patients were followed-up, and prognosis at a specific period was observed in our study. However, shortcomings should also be considered. Major limitations of this study include that the study is a single-center, small-sample study; we look forward to the production of multicenter, large-sample data for further confirmation. In addition, there are some deficiencies in this technique. For example, although ESAT-6 is absent in all BCG strains and most environmental mycobacteria, the positive results of the ELISPOT assay may be caused by the infection of *Mycobacterium* kansaii, *Mycobacterium simiae*, *Mycobacterium gordonae* or *Mycobacterium marinum*. It should be screened in clinical diagnosis.

## Conclusions

In conclusion, the IFN-gamma release test can be used for the early diagnosis of CAPD-related TBP; compared with peripheral blood, peritoneal dialysate may be a more effective and accurate medium to diagnose CAPD complicated with tuberculous peritonitis.
